# Simultaneous alcohol and cannabis expectancies predict simultaneous use

**DOI:** 10.1186/1747-597X-1-29

**Published:** 2006-10-11

**Authors:** Sara Smucker Barnwell, Mitch Earleywine

**Affiliations:** 1 VA Greater Los Angeles Healthcare System (GLA), Mail Code 116B, 11301 Wilshire Blvd. Los Angeles, CA, 90073, USA; 2 University at Albany, State University of New York, Department of Psychology, Social Sciences 369, 1400 Washington Ave, Albany, NY 12222, USA

## Abstract

**Background:**

Simultaneous use of alcohol and cannabis predicts increased negative consequences for users beyond individual or even concurrent use of the two drugs. Given the widespread use of the drugs and common simultaneous consumption, problems unique to simultaneous use may bear important implications for many substance users. Cognitive expectancies offer a template for future drug use behavior based on previous drug experiences, accurately predicting future use and problems. Studies reveal similar mechanisms underlying both alcohol and cannabis expectancies, but little research examines simultaneous expectancies for alcohol and cannabis use. Whereas research has demonstrated unique outcomes associated with simultaneous alcohol and cannabis use, this study hypothesized that unique cognitive expectancies may underlie simultaneous alcohol and cannabis use. Results: This study examined a sample of 2600 (66% male; 34% female) Internet survey respondents solicited through advertisements with online cannabis-related organizations. The study employed known measures of drug use and expectancies, as well as a new measure of simultaneous drug use expectancies. Expectancies for simultaneous use of alcohol and cannabis predicted simultaneous use over and above expectancies for each drug individually.

**Discussion:**

Simultaneous expectancies may provide meaningful information not available with individual drug expectancies. These findings bear potential implications on the assessment and treatment of substance abuse problems, as well as researcher conceptualizations of drug expectancies. Policies directing the treatment of substance abuse and its funding ought to give unique consideration to simultaneous drug use and its cognitive underlying factors.

## Background

Expectancies represent intervening cognitive variables that connect memory and behavior, and reflect knowledge of a relationship between events and objects [1]. Bolles [2] identified expectancies as environmental stimulus-outcome contingencies that directly affect behavior, and regarded expectancies as synonymous with the concept of association. Expectancies represent individual learning associations made between stimuli, individual responses and resulting outcomes. Heavy drug consumers may be more likely to activate positive expectancies for drug effects [e.g. [3]].

While researchers have investigated the cognitive mechanisms underlying single substance use [e.g. [4]], a relative dearth of research exists on the cognitive mechanisms informing simultaneous polydrug use or their role in drug abuse. Research suggests that similar mechanisms may underlie expectancies for both alcohol and cannabis use. Stacy [5] demonstrated that similar memory association mechanisms underlie both alcohol and cannabis use. Boys and Marsden [6] found that polysubstance users' expectations regarding relief of negative mood states increased the likelihood to use drugs such as cannabis and alcohol simultaneously. Yet no studies assess simultaneous drug use expectancies, despite findings that simultaneous use of cannabis and alcohol may yield different outcomes than use of cannabis alone [e.g. [6]].

Researchers identify cannabis and alcohol as the two substances most frequently used simultaneously [7,8]. The simultaneous use of alcohol and cannabis may introduce greater problems than the use of both drugs independently or even concurrently. Staines et al [9] reported a positive relationship between problems with alcohol and simultaneous use of illicit drugs such as cannabis. Other studies link simultaneous cannabis and alcohol use with increased negative consequences such as psychological distress, psychopathology [10] and substance dependence [9].

Earleywine and Newcomb [7] distinguished between concurrent drug use (e.g. use on separate occasions) and simultaneous use of multiple drugs, and found the two types of use form two distinct constructs. Simultaneous polydrug users may experience greater psychological distress and other negative consequences associated with drug use compared to other substance users [11,12]. Smucker Barnwell et al [13] found that interactions between measures of cannabis use and alcohol consumption significantly predicted cannabis dependence among frequent cannabis users. Stenbacka [14] found that simultaneous use of alcohol and cannabis among adolescents predicted later problems with either drug. Alcohol use and abuse serve as primary predictors of cannabis dependence [15-17], and cannabis dependence covaries more with alcohol dependence than with most other psychiatric diagnoses [18]. Similarly, several studies find that cannabis use frequently occurs among individuals with alcohol dependence diagnoses [18]. Heavy alcohol consumption among cannabis users may result in more problematic cannabis use, less successful cessation and more resulting negative life consequences [19].

The present research also sought to understand the etiology underlying the increased risks associated with simultaneous polydrug use. The interaction of alcohol and the chemical component known to cause the majority of cannabis' intoxicating effects, delta-9 tetrahydrocannabinol (THC), may offer a pharmacological explanation. Lukas and Orozco [20] found that smoking cannabis while also consuming ethanol lead to increased THC levels in the blood and more intensely positive reported subjective effects. Individuals who consume both drugs simultaneously may experience higher absorption rates of THC, increased positive effects of the drug, and, perhaps, greater cannabis or even alcohol dependence symptoms. Outside of pharmacological explanations, researchers posit that expectancies play a major role in the prediction of substance use and abuse [e.g. [5]].

Whereas different behavioral outcomes are predicted for simultaneous use of cannabis and alcohol, it seems plausible that unique cognitive expectancies underlie these outcomes. Individuals using several drugs simultaneously seem likely to possess different expectancies than an individual using a single substance. Perhaps these individuals possess unique cognitive templates that inform simultaneous polydrug use. Beyond individual drug expectancies, this study sought to identify unique cognitive constructs motivating simultaneous drug use. Perhaps simultaneous alcohol and cannabis use possess a distinct and unique set of expectancies, predicting simultaneous use beyond individual drug expectancies. This study examined whether simultaneous alcohol and cannabis use expectancies more accurately predicted simultaneous cannabis and alcohol use than single substance expectancies alone. Since little research exists on the identification and measurement of simultaneous drug use expectancies, this project offers a unique contribution to the existing literature on substance use and its motivators. Researchers and policy makes alike stand to benefit from greater understanding of usual patterns of drug use, their precipitating factors and, by extension, pathways to intervention and even prevention.

## Results

### Participants

Members of the Marijuana Policy Project (MPP) and National Organization for the Reform of Marijuana Laws (NORML) listserves received an email requesting survey participation. The study focused on individuals consuming cannabis and alcohol at least once a month to ensure sufficient information regarding target behaviors (N = 2637). Two thirds of respondents were male (66%); one third was female (34%). Respondents ranged in age from 13 to 86. Their mean age was 34.0 years (SD = 13.3). The average respondent first tried cannabis at the age of 16.0 years (SD = 3.9). The group was primarily of European descent, with other ethnicities ranking far behind. The majority of respondents possessed a Bachelor's degree, Associate's degree, or some college credit completion. The average participant earned an annual income of less than $40,000 (See Table 1). All participants completed an online consent form in accordance with university ethical procedures. To ensure participant confidentiality, respondents were not required to provide any identifying information. Those participants who wished to enter into a raffle for one of several $100 gift certificates could provide an email address. Email addresses were immediately disconnected from all participant data to ensure respondent anonymity.

**Table 1:** Demographics as measured by frequencies

**Ethnic Group (N = 2580)**	
European descent	91%
Latino	4%
African descent	2%
Asian descent	1%
Mixed ethnicity	1%
Other ethnic groups (e.g. Native American, Middle Eastern)	1%

**Highest Level of Education (N = 2635)**	

Some high school completed	4%
High School diploma	11%
Some college credit completed	39%
Associate's degree	12%
Bachelor's degree	23%
Master's degree	7%
Degree beyond Master's training	4%

**Income Range (N = 2529)**	

Less than $20,000 per year	35%
$20,000–$40,000	29%
$40,000–$60,000	16%
$60,000–$80,000	7%
$80,000–$100,000	4%
$100,000+	4%

### Alcohol consumption measures

The average respondent consumed 44.2 drinks per month (SD = 55.4) when consuming any alcohol. This score derived from a measure inquiring about average incidents of drinking per month, and usual numbers of drinks per incident. When drinking alcohol and consuming cannabis together within three hours of each other (e.g. only alcohol and cannabis), the average participant consumed fewer (M = 28.7; SD = 49.4) drinks per month. In contrast, when drinking only alcohol (e.g. with no other substances), respondents reported drinking still fewer (M = 20.5; SD = 42.3) drinks per month (See Table 2). Respondents reported different amounts of alcohol when drinking only alcohol, alcohol with cannabis and alcohol with any other substance (See Table 3).

**Table 2:** Alcoholic Drinks Per Month and Ounces Cannabis Consumption Overview

**Alcohol/Cannabis**	**Mean**	**SD**	**Range**
All Alcohol Per Month (N = 2618)	M = 42.4	SD = 55.4	.5 – 625 drinks
Only Alcohol Per Month (N = 2594)	M = 20.5	SD = 42.3	0 – 620 drinks
Simultaneous Drinks Per Month (N = 2744)	M = 28.7	SD = 49.4	0 – 600 drinks
All Cannabis Per Month (N = 2627)	M = 2.8	SD = 1.5	< 1/4 oz – >1 oz (1–5)
Only Cannabis Per Month (N = 2591)	M = 2.6	SD = 1.3	< 1/4 oz- >1oz (1–5)
Simultaneous Cannabis Per Month (N = 2577)	M = 1.9	SD = 1.3	< 1/4 oz – >1 oz (1–5)

Note: Metric weight of cannabis measured on 5-point scale (1 = <1/4 oz; 2 = 1/4–1/2 oz; 3 = 1/2–3/4 oz; 4 = 3/4–1 oz; 5 = >1 oz).

**Table 3:** Paired T-tests comparing drug use

**Alcohol/Cannabis Pairs**	**Mean**	**SD**	**Relevant t values**
All Alcohol Per Month	M = 42.4	SD = 55.4	t(2573) = 24.0**
Only Alcohol Per Month	M = 20.47	SD = 42.3	
All Alcohol Per Month	M = 42.4	SD = 55.4	t(2574) = 16.3**
Simultaneous Drinks Per Month	M = 28.7	SD = 49.4	
Only Alcohol Per Month	M = 20.47	SD = 42.3	t(2558) = 8.1**
Simultaneous Drinks Per Month	M = 28.7	SD = 49.4	
All Cannabis Per Month	M = 2.8	SD = 1.5	t(2585) = 3.4**
Only Cannabis Per Month	M = 2.6	SD = 1.3	
All Cannabis Per Month	M = 2.8	SD = 1.5	t(2572) = 32.4**
Simultaneous Cannabis Per Month	M = 1.9	SD = 1.3	
Only Cannabis Per Month	M = 2.6	SD = 1.3	t(2543) = 29.4**
Simultaneous Cannabis Per Month	M = 1.9	SD = 1.3	

*Note*. * *p *< .05. ** *p *< .01

### Cannabis consumption measures

Respondents used cannabis in any combination (e.g. alone, with other substances) an average of 22.4 days per month (SD = 10.3). They consumed approximately three quarters of an ounce of cannabis per month (2.8 on a 5-point scale; SD = 1.5, < 1/4 ounce). When using only cannabis, they used cannabis fewer (M = 19.1; SD = 10.8) days per month, and tended to consume less cannabis (M = 2.6, > 1/2 oz; SD = 1.4 < 1/4 oz). When using cannabis and alcohol together within three hours of each other, however, respondents consumed cannabis the fewest (M = 7.8; SD = 8.6) days per month and in the least amounts (M = 1.9, about 1/4 ounce; SD = 1.3, < 1/4 oz) (See Tables 4 and 5).

**Table 4:** Other Polydrug Consumption as measured by frequency (N = 2637)

**Other Drug Combinations**	
Marijuana and hallucinogens	25%
Marijuana with sedatives	17%
Alcohol with hallucinogens	14%
Alcohol with sedatives	14%
Marijuana with cocaine	13%
Marijuana with stimulants	13%
Alcohol with Cocaine	12%
Alcohol with stimulants	11%
Stimulants with sedatives	4%
Cocaine with other stimulants	3%
Cocaine with sedatives	3%
Hallucinogens with sedatives	3%
Cocaine with hallucinogens	1%

**Table 5:** Bivariate correlations among Indicators of Cannabis and Alcohol Use

**Measure**	**1**	**2**	**3**	**4**	**5**	**6**	**7**	**8**	**9**	**10**	**11**
**1. Gender**	_										
**2. Age**	.02	_									
**3. All Drinks/Month**	.11**	-.09**	_								
**4. Only Drinks/Month**	.08**	-.11**	.55**	_							
**5. Drinks/Can Month**	.06**	.09**	.68**	.26**	_						
**6. All Cann/Month**	-.01	-.13**	.08**	-.13**	.20**	_					
**7. Cann Only/Month**	-.01	-.15**	.02	-.11**	.14**	.91**	_				
**8. Cann/Drinks Month**	-.02	-.01	.20	-.03	.31**	.60**	.56**	_			
**9. Alcohol Expectancies**	.06**	-.22**	.26**	.20**	.22**	.01	-.01	.05*	**_**		
**10. Cann Expectancies**	-.02	-.04*	-.02	-.09**	.04*	.23**	.24**	.14**	.05*	_	
**11. Simult Expectancies**	.07**	-.13**	.18**	.05*	.25**	.11**	.08**	.15**	.53**	.43**	**_**

*Note*. * *p *< .05. ** *p *< .01; All Drinks/Month = Number alcoholic drinks consumed in the month; Only Drinks/Month = Number alcoholic drinks consumed when *only *drinking alcohol (e.g. no other substances); Drinks/Cann Month = Number alcoholic drinks consumed when also using cannabis; All Cann/Month = Metric weight of any cannabis used in the month; Cann Only/Month = Metric weight of cannabis consumed when only using cannabis; Cann/Drinks Month = Metric weight of cannabis consumed when also drinking alcohol in the month; Simult Expectancies = Combined simultaneous expectancy score.

### Simultaneous consumption measures

Participants then completed the Simultaneous Polydrug Use Questionnaire [DUQ; 21]. The DUQ assessed frequency of use of alcohol/drug and drug/drug combinations among seven classes of drugs: alcohol, cannabis, cocaine, opiates, sedatives, stimulants and hallucinogens. The use of this measure permitted the researcher to examine patterns of simultaneous drug use outside of cannabis and alcohol combinations. Simultaneous use was defined as use of two or more substances occurring within three hours of each other. Approximately one quarter of respondents reported consuming drug combinations other than alcohol and cannabis.^1 ^Removal of participants using drug combinations other than alcohol and cannabis did not alter findings. The majority of these respondents reported consuming marijuana with hallucinogens at least once a month in the past four-months. Other drug combinations were significantly less common (See Table 4).

### Explicit measures of alcohol expectancies

Participants completed subscales of the Alcohol Expectancy Questionnaire [AEQ; 22], a widely used 120 item self-report questionnaire for measuring alcohol expectancies. The scale demonstrates predictive and concurrent validity and is the most commonly used measure for alcohol expectancies. The AEQ consists of six subscales: global positive changes, sexual enhancement, social and physical pleasure, social assertiveness, relaxation, and arousal/aggression. To reduce participant burden, the study employed only global positive changes and relaxation subscales. Consistent with the measure's intent, we altered the measure instructions to indicate that items referred to the effects of alcohol use only. The measure demonstrated high internal consistency as measured by Cronbach's alpha (α = .89). Respondents endorsed 13.7 (SD = 7.4) alcohol expectancy items out of a possible score of 37, indicating moderate expectancies regarding the positive effects of alcohol.

### Explicit measures of cannabis expectancies

The Marijuana Effect Expectancy Questionnaire [MEEQ; 23] measured explicit expectancies regarding cannabis consumption. The complete measure consists of 78 true/false items and six subscales: (a) cognitive and behavioral impairment, (b) relaxation and tension reduction, (c) social and sexual facilitation, (d) perceptual and cognitive enhancement, (e) global effects, and (f) craving and physical effects. To reduce participant burden, the study employed only the relaxation and tension reduction subscale and global effects subscale. Altered measure instructions clarified that the questions referred to the effects of only cannabis use. The relaxation (α = .86) and tension reduction subscales (α = .72) each demonstrated sound internal consistency in the sample. Analyses employed a combined expectancy score. Respondents endorsed 15.6 (SD = 2.3) cannabis expectancy items out of a total of possible score of 19, indicating high expectancies regarding positive effects of cannabis.

### Explicit measure of simultaneous alcohol and cannabis use expectancies

Using the Alcohol Expectancy Questionnaire (AEQ) and the Marijuana Effect Expectancy Questionnaire (MEEQ), the researcher developed a measure of expectancies for simultaneous cannabis and alcohol use. First, the measure repeated all previous AEQ items. If participants endorsed the item, an additional question followed: "How does marijuana alter this effect?" The measure employed a Likert-type scale ranging from -3 (Make it less intense) to 3 (Makes it more intense). Thus respondents reported how alcohol impacted their experiences, and then indicated if simultaneous cannabis and alcohol impacted their experiences differently. Next, items from the MEEQ subscales also repeated. Again, if subjects endorsed the item, the question "How does alcohol alter this effect?" appeared. Thus respondents indicated how cannabis impacted them, and indicated if simultaneous cannabis and alcohol impacted them differently. Total scores were derived by assigning natural number analogs to scale value (e.g. 1–7), and summing the scores. Items not endorsed the second administrations of the AEQ and MEEQ were not given the follow-up question (e.g. "How does alcohol/cannabis alter this effect" and were thus not included in their respective summary scores for simultaneous expectancies. We believe that this scoring method is approximately consistent with the original expectancy measure scoring which excludes items not endorsed from total expectancy scores. Lower scores indicated a lesser belief that simultaneous use intensified the experience, while higher numbers indicated a greater belief that simultaneous use intensified drug use experiences.

After several phases of revisions, pilot group participants ultimately indicated that the measure was clear and comprehensible. The first administration of the AEQ correlated very highly, but not perfectly with the second administration (N = 2635, r = .85, p < .001). The second administration of the MEEQ correlated only moderately with the first administration (N = 2634, r = .44, p < .001). The measure demonstrated sound internal consistency in the online sample (α = .81). Scores were derived from respondents' report of how one substance impacted the intensity of the other on the 7-point scale. Higher scores indicated increased belief that simultaneous use of the two substances made the experience more intensely positive. The average respondent reported a low to moderate simultaneous expectancy score (M = 89.6; SD = 51.6). Summed, standardized component scores served as an index of total expectancies that consumption of one substance would alter experiences with the other.

### Correlations

Large, significant correlations emerged between the various measures of alcohol consumption. Similarly, significant correlations emerged among measures of cannabis consumption. Cannabis and alcohol expectancy scores demonstrated small but significant correlations with alcohol and cannabis consumption measures (See Table 5).

### Regressions

The first linear regression equation examined alcoholic drinks consumed per month when also using cannabis as the dependent variable. Lognormal transformations improved the skew of the dependent variable [24]. The skewness and kurtosis statistics of the measure were 4.3 and 27.4, respectively, prior to lognormal transformations and -.03 and -.91 afterwards. Age and gender acted as covariates. Alcohol expectancies (e.g. AEQ), cannabis expectancies (e.g. MEEQ) and simultaneous alcohol and cannabis expectancies each acted as predictors. Age and alcohol expectancy scores each demonstrated a main effect. In addition, simultaneous expectancies for alcohol and cannabis demonstrated a main effect. As predicted in study hypotheses, simultaneous expectancies predicted simultaneous alcohol and cannabis use beyond individual drug expectancies (See Table 6).

**Table 6:** Linear regression: simultaneous alcohol and cannabis expectancies account for variance in the prediction of simultaneous alcohol and cannabis use beyond individual alcohol expectancies or cannabis expectancies.

**Variable**	Δ**R**^2^	**R**^2^	β	**t**
*Simultaneous Alcohol and Cannabis Use Drinks Per Month (Log Normal; df = 2517)*				
Age	.005		-067	-3.66**
Gender	.001	.006	.036	1.87
AEQ	.008	.014	.167	4.85**
MEEQ	.004	.018	.065	3.39**
Simultaneous Expectancies	.020	.038	.185	7.60**
				
*Simultaneous Alcohol and Cannabis Use Cannabis Per Month (Log Normal; df = 2552)*				
Age	.019		-.143	-7.12**
Gender	.000	.019	-.021	-1.09
AEQ	.002	.021	-.066	2.43*
MEEQ	.001	.022	.026	1.34
Simultaneous Expectancies	.003	.025	.069	2.59*

*Note: *Standardized regression coefficients (βs) derive from the step in which they are added to the equation.

*p < .05 **p < ..01 _p = .05 Cannabis per month with alcohol = Metric weight of cannabis consumed when also drinking alcohol. Drinks per month with Cannabis = Number of alcoholic drinks when also consuming cannabis AEQ = Alcohol Expectancies; MEEQ = Cannabis Expectancies.

In the second linear regression equation, metric weight of cannabis consumed per month when also using alcohol served as the dependent variable. Again, lognormal transformations improved the skew of the dependent variable [24], and age and gender acted as covariates. The skewness and kurtosis statistics were 1.3 and .36, respectively, before lognormal transformations and .98 and -.52 afterwards. Cannabis expectancies, alcohol expectancies and simultaneous expectancies again acted as independent variables. Only age and simultaneous expectancy scores demonstrated main effects in the prediction of metric weight of cannabis consumed when also drinking. Thus simultaneous expectancies again accounted for unique variance beyond individual drug expectancy scores. Age again demonstrated a significant effect (See Table 6). It is notable that the authors examined the same equations using only measures of use frequency (e.g. days per month), and encountered no different findings.

A third equation examined frequency of simultaneous use. Days per month in which respondents used alcohol and cannabis simultaneously served as the dependent variables. Again, age, gender, individual alcohol expectancies, individual cannabis expectancies and simultaneous expectancy scores each acted as predictors. Age and cannabis expectancies each predicted simultaneous use. Again, simultaneous expectancies predicted simultaneous use beyond variance accounted for by individual drug expectancy measures (See Table 7). In this equation, unique variance accounted for simultaneous expectancies was notably larger than that of individual drug expectancies.

**Table 7:** Linear regression: simultaneous alcohol and cannabis expectancies account for variance in the prediction of days per month using alcohol and cannabis simultaneously beyond individual alcohol expectancies or cannabis expectancies.

**Variable**	Δ**R**^2^	**R**^2^	β	**t**
*Days Using Alcohol and Cannabis Simultaneously (df = 2539)*				
Age	.014		.123	6.21**
Gender	.001	.015	-.028	-1.42
AEQ	.002	.017	.049	1.84
MEEQ	.001	.018	.046	2.39*
Simultaneous Expectancies	.017	.035	.179	6.74**

*Note: *Standardized regression coefficients (βs) derive from the step in which they are added to the equation.

*p < .05 **p < .01 _p = .05 Cannabis per month with alcohol = Metric weight of cannabis consumed when also drinking alcohol. Drinks per month with Cannabis = Number of alcoholic drinks when also consuming cannabis AEQ = Alcohol Expectancies; MEEQ = Cannabis Expectancies.

## Discussion

In this study, simultaneous expectancies predicted simultaneous drug use quantity and frequency. Simultaneous expectancies demonstrated the strongest main effect in the prediction of number of days consuming alcohol and cannabis simultaneously, as well as the number of alcoholic drinks when also consuming cannabis. Age, alcohol expectancies and simultaneous expectancies predicted amount of cannabis consumed when drinking alcohol.

### Effect size and power

Given the vast sample size, a potential limitation was the clinical significance of effect size. Perhaps the large sample size provided sufficient statistical power to reveal relatively small findings with marginal clinical significance. It is plausible that the extremely large online data sample size may have resulted in the magnification of findings that bear little real-world relevance. Yet effects are frequently difficult to encounter in drug use data [e.g. [13]]. Although the effects associated with simultaneous expectancies are small and do not consistently account for the largest measures of unique variance in the regression equations, the existence of statistically significant effect merits further examination of the potential clinical significance of these findings.

### Homogeneity of socio-economic status and ethnicity

Participants were largely Caucasian, educated and of moderate socioeconomic status. Low socioeconomic status [25] and low educational attainment [26] both correlate with drug use in previous research. Individuals with lower income or education may have evidenced a positive link between single substance expectancies and use regardless of simultaneous alcohol consumption. A more ethnically diverse sample may have demonstrated different findings as well.

### Sample selection biases

Participants belonged to the National Organization for the Reform of Marijuana Laws or the Marijuana Policy Project. Individuals in this group were more likely to be aligned with the organization's mission of cannabis activism. Individuals belonging to these organizations may have been more likely to report positive associations with cannabis, accounting for higher cannabis expectancies in the online data sample. Also, participants belonging to these organizations may have been less likely to report problem-inducing cognitions associated with cannabis. We know of no research that would indicate that minimizing of cannabis problems would impact reports of expectancies. Respondents continued to report considerable use. Previous studies have successfully identified alcohol and cannabis problems in similar online populations [see [13]]. Thus this potential limitation does not represent an insurmountable barrier to data interpretation. These findings may offer greatest importance for those individuals who are frequent and/or heavy users of cannabis and alcohol. As previously mentioned, a more ethnically diverse sample would have offered more generalizable findings. Still, whereas the present sample provided a cross-section of individuals who consume the two drugs and demonstrate associated problems, we contend that these data offer some generalizability for individuals who consume cannabis and alcohol simultaneously.

### Measurement

Whereas this study established a new measure of simultaneous alcohol and cannabis expectancies, it is possible that this measure introduced problems to the study. High disagreement between the first and second administration of items used in the MEEQ could indicate a problem with the measure. Reordering of items (e.g. MEEQ before AEQ) could significantly alter measure scores. Inherently, the simultaneous expectancy measure was lengthy and repetitious. It is possible that some participants encountered the measure as burdensome. During pilot testing, respondents indicated that although the measure was long and repetitious, these features did not preclude accurate completion of the survey. However, it is possible that the actual study respondents encountered the measure differently than pilot test participants.

Furthermore, the current administration of the simultaneous expectancies measure is likely the first in an iterative development process. The creation of a new measure typically requires numerous administrations and intensive research into the measure validity and reliability. Further research geared toward developing a more comprehensive measure of simultaneous alcohol and cannabis expectancies could provide greater evidence regarding unique expectancies for increased drug effects.

Also, issues of self-report may have obstructed findings in these data. Although data collection was anonymous, some participants may have encountered self-presentational issues that prevented them from reporting substance use. Although past research suggested that online measures elicited greater reports of drug consumption compared to laboratory-based measures [27], respondents may have been hesitant to report drug use and especially illicit drug use. It is unclear whether those who suffer the worst negative consequences of drugs are willing to disclose all symptoms and the full extent of their drug use in an Internet context. Still, the aforementioned limitations are common to many studies on these topics [see [28]], and may not present insurmountable barrier to interpretation of the data.

### Experimental manipulation

As with any moderator that has not been manipulated experimentally, findings in this study could have arisen from drug use correlates rather than substance use itself. Personality traits, family history, genetics, or a plethora of other factors that correlate with alcohol and cannabis use may have actually served as the impetus for simultaneous alcohol and cannabis use. For example, sensation seekers may be more likely to score higher on drug expectancy measures and engage in more polydrug use. Perhaps sensation seeking, a known correlate of cannabis use [29], or its genetic correlates were actual factors underlying simultaneous alcohol and cannabis use. Nevertheless, the idea that unique expectancies regarding simultaneous use leads to increased simultaneous polydrug expectancies seems tenable.

### Implications

Whereas the concept of simultaneous drug expectancies is a relatively new adaptation of the existing literature on expectancies, additional study could assist researchers in the development of this potentially useful research construct and measurement tool. The second administration of MEEQ items came toward the end of the study measures. Perhaps moderate correlations between first and second administrations of MEEQ items suggest participant fatigue toward the end of the study. Future studies may lessen participant burden by abbreviating the scale or dividing test administration into two sessions. Examination of other relevant subscale items from the AEQ and MEEQ may merit further exploration. The recommended procedure of reverse scoring negative items in the MEEQ may not yield items comparable to positive items on the AEQ. That is, the reverse score of a negative statement may not be the ideological analog to a positive statement. Future research may wish to explore rewriting items from the MEEQ's global scale under the supervision of the scale's authors.

Finally, although a combined expectancy score provided superior predictive powers than its component scales (e.g. alcohol alters experiences with marijuana; marijuana alters experiences with alcohol) in this study, future researchers may wish to examine these components separately. Perhaps further study will reveal different outcomes associated with the belief that one substance impacts the other to a greater extent.

## Conclusion

If simultaneous expectancies offer a sound predictor of simultaneous drug use and, ultimately, problems, researchers may wish to integrate these findings into drug treatment and prevention efforts, including intervention attempts to decrease positive drug expectancies [e.g. [30,31]]. Research examining specific expectancies (e.g. simultaneous alcohol and cannabis expectancies for aggression) could explore simultaneous expectancies' capacity to predict particular simultaneous drug use consequences (e.g. drug-related violence).

## Authors' contributions

**SSB: **1) Designed study, acquired data, analyzed data, and interpreted findings. 2) Involved in composition of manuscript and its revisions and 3) has given final approval of the version to be published. This author obtained funding from the Kellerman Research Fund at the University of Southern California to support this project.

**ME: **Advised and oversaw in study design, data analyses and interpretation of findings. 2) Reviewed manuscript, and made substantive edits throughout revision process 3) has given final approval of the version to be published. This author obtained permission from MPP and NORML to use their mailing lists.

## Note

^1 ^Removal of participants using drug combinations other than alcohol and cannabis did not alter findings.
